# Morphological stabilization and KPZ scaling by electrochemically induced co-deposition of nanostructured NiW alloy films

**DOI:** 10.1038/s41598-017-18155-7

**Published:** 2017-12-21

**Authors:** P. A. Orrillo, S. N. Santalla, R. Cuerno, L. Vázquez, S. B. Ribotta, L. M. Gassa, F. J. Mompean, R. C. Salvarezza, M. E. Vela

**Affiliations:** 1 INQUINOA-CONICET, Instituto de Química Física, Facultad de Bioquímica, Química y Farmacia, Universidad Nacional de Tucumán, Ayacucho 471, (4000) San Miguel de Tucumán, Argentina; 2 Departamento de Física and Grupo Interdisciplinar de Sistemas Complejos, (GISC), Universidad Carlos III de Madrid, Avenida de la Universidad 30, 28911 Leganés, Spain; 3 Departamento de Matemáticas and GISC, Universidad Carlos III de Madrid, Avenida de la Universidad 30, 28911 Leganés, Spain; 4 Materials Science Factory, Instituto de Ciencia de Materiales de Madrid (CSIC), 28049 Madrid, Spain; 5 Instituto de Investigaciones Fisicoquímicas Teóricas y Aplicadas (INIFTA), Universidad Nacional de La Plata - CONICET-, Sucursal 4 Casilla de Correo 16, (1900) La Plata, Argentina

## Abstract

We have assessed the stabilizing role that induced co-deposition has in the growth of nanostructured NiW alloy films by electrodeposition on polished steel substrates, under pulsed galvanostatic conditions. We have compared the kinetic roughening properties of NiW films with those of Ni films deposited under the same conditions, as assessed by Atomic Force Microscopy. The surface morphologies of both systems are super-rough at short times, but differ at long times: while a cauliflower-like structure dominates for Ni, the surfaces of NiW films display a nodular morphology consistent with more stable, conformal growth, whose height fluctuations are in the Kardar-Parisi-Zhang universality class of rough two-dimensional interfaces. These differences are explained by the mechanisms controlling surface growth in each case: mass transport through the electrolyte (Ni) and attachment of the incoming species to the growing interface (NiW). Thus, the long-time conformal growth regime is characteristic of electrochemical induced co-deposition under current conditions in which surface kinetics is hindered due to a complex reaction mechanism. These results agree with a theoretical model of surface growth in diffusion-limited systems, in which the key parameter is the relative importance of mass transport with respect to the kinetics of the attachment reaction.

## Introduction

The electrodeposition of metallic alloys on steels has become a widely used procedure to improve the corrosion resistance, as well as the mechanical and tribological properties in the technological applications of these materials. Chemical, physical, and mechanical properties can be controlled by understanding how the material is being formed at the atomic level^1,2^. Electrodeposition of alloys requires simple equipment, compared with other techniques —like chemical or physical vapor deposition, or sputtering—, and allows for the precise control of composition, thickness, and structure of the deposit, and for the possibility to cover complex surfaces topographies^3–6^. In particular, tungsten alloys containing Ni, Co, and Fe have been subject of investigation due to their high hardness and abrasion resistance, high melting temperature, and corrosion behavior^5–11^. Tungsten cannot be electrodeposited from aqueous solutions of its salts, but it can be co-deposited with iron group metals such as Fe, Co, and Ni^5^. Thus, Ni-W alloys exhibit higher thermal stability than pure Ni, and excellent magnetic, electrical, and tribological properties^5^. Furthermore, they have high abrasion resistance and their hardness is three times higher than that of electrodeposited Ni^12^. In addition, their resistance to strong oxidizing acids makes Ni-W alloys particularly attractive. For example, the corrosion rate of an amorphous Ni–W deposit layer in HCl acid at 30 °C is only 1/40th and in NaCl solution is 1/3 of its value for 304 Stainless Steel^13^. Likewise, in neutral electrolytes containing sulfate anions, Ni-W coatings protect the carbon steel substrate from pitting^14,15^. This set of properties makes Ni-W alloys a very interesting material for many technological applications, such as protective coatings of copper and steels, in cutting tools, magnetic heads and relays, high-strength wires, and springs, among others^5^. They are also constituents of electrodes for hydrogen electrocatalysis^16^ and substituents for hard chromium plating in aerospace industry^17^.

In general, understanding the processes and physical mechanisms controlling the morphology and roughness of the film surfaces is very important both from the fundamental and from the applied points of view^18–20^. For instance, nanoscale surface structure and grain (crystallite) size play relevant roles in materials performance^21^. Furthermore, the surface roughness plays a key role in many applications and properties such as contact mechanics, sealing, friction, adhesion, and wettability^18,22^. It is also very relevant to the final surface gloss^23,24^ and to resistance to corrosion, whose onset is facilitated by grooves and defects^21^. Roughness properties are becoming even more relevant recently, since many new devices and tools require control of the film structure and morphology even down to the atomic scale^25^. Hence, the analysis of the surface morphology evolution with growth time within the framework of kinetic roughening and associated scaling concepts has proved adequate to determine the main physical mechanisms controlling the films surface morphology when it is strongly disordered^26–28^. Out-of-equilibrium surfaces are said to be kinetically rough when roughness fluctuations follow characteristic (power) laws with time and length scales, and have been successfully assessed in thin film growth, both of organic^29,30^ and of inorganic materials. Among the last group, the main mechanisms governing the surface roughening of films grown by different deposition techniques have been identified, including e.g. physical evaporation^31^, sputtering^32–34^, chemical vapor deposition (CVD)^35,36^, chemical bath^37^, or Molecular Beam Epitaxy^38^. From a basic point of view, some kinetically rough systems are proving themselves as a paradigm of non-equilibrium fluctuations. This is the case for those in the so-called Kardar-Parisi-Zhang (KPZ) universality class^39^. Driven by breakthroughs on the exact solution of the KPZ equation and related growth models (for reviews, see e.g.^40,41^), that have been experimentally validated for one^42–45^ and two-dimensional^46,47^ interfaces, this class is recently focusing an enormous attention. Indeed, it is being found to encompass the scaling behavior of many fluctuating 1D systems, from classical non-linear oscillators^48^ to superfluids^49^, or Fisher waves^50^. Remarkably, beyond critical exponents values, universal KPZ scaling also applies to the probability distribution of height fluctuations. This is notably provided by the Tracy-Widom distribution of the largest eigenvalue of Hermitian random matrices^40,41,51^ (resp. suitable generalizations thereof^52,53^) for 1D (resp. 2D) interfaces.

Films grown by electrochemical deposition (ECD) have also been studied under the kinetic roughening framework^54–58^. This deposition technique is characterized by a growth dynamics that results from the competition between diffusive transport of the metal ions through the electrolyte to reach the film surface and the kinetics of their eventual attachment^59,60^. Generally, the former is the source of a morphological instability since any surface protrusion, initially originated by the random arrival of the metal ions, will be amplified by the higher probability for the ions to attach to more prominent surface features. Moreover, this local growth probability depends on the detailed surface morphology throughout the film surface. E.g., a large mound close to a surface depression shields the latter from the arrival of depositing species. This turns ECD into a non-local process. This situation usually leads to the development of rough surfaces and anomalous scaling behavior, see below. However, organic additives usually reduce the roughening process and lead to substantially smoother films^61^. Most of these studies have been carried out under direct current (DC) ECD. In contrast, only one of them has been performed using a pulsed current (PC) ECD technique, to deposit Ni films^55^. Although the dynamic scaling analysis of Ni films grown under DC and PC conditions did not show great differences, the PC configuration may influence the film morphology^62^. Moreover, under PC conditions where an effective current is applied during a time, *t*_ON_, and no current circulates during a relaxation time, *t*_OFF_, uneven deposits caused by mass transport and hydrogen evolution processes are minimized, as are local pH changes.

Although many alloys can be directly deposited, some elements like Mo, Ge, P, and W cannot be deposited as such from aqueous solutions of their compounds. However, they can be co-deposited with iron-group metals. The term “induced co-deposition” was introduced by Brenner^4^ to characterize the process in which a metal that cannot be deposited pure from aqueous solution is deposited with another metal forming an alloy. It could be considered anomalous since the composition of the alloy cannot be predicted from the electrochemical properties of each component. Frequently the induced co-deposition takes place using a complexing agent (most often, citrate) in the plating bath that allows to approximate the electrode potential of the metals to be electrodeposited. The ions of the more noble metal, once coordinated into the complex, change their tendency to be reduced, which enables their co-deposition with the less-noble (more active) metal. The solution chemistry can still be quite convoluted since complexes of each metal can be formed with the ligand and with ligands containing both metals.

In this work, we study the surface growth dynamics of NiW alloys on steel substrates obtained by electrochemical induced co-deposition. To our knowledge, this is the first study addressing the full growth dynamics that occurs under this technique. In order to better assess the characteristics of this method, we have also studied the electrodeposition of Ni films on steel substrates, using baths containing citrate as a complexing agent in all cases. We made this choice, first because there are already similar studies in the literature on metal film growth by direct ECD using a metal salt^54–56^. Second, because by studying both systems we can discriminate between the effects of the citrate complexing agent and those due to induced co-deposition itself. We have performed the electrodepositions using the PC mode in both cases. This configuration adds more novelty to the study since only one previous work has dealt with the growth dynamics of metal films electrodeposited under PC conditions^55^. Moreover, in spite of the complexities of the present technique, our results can be rationalized in terms of a rather general model of the competition between diffusion-limited instabilities and the stabilizing role of finite surface kinetics^59,60^, which accounts for the prevalence of the celebrated KPZ scaling under working conditions with inefficient surface attachment.

## Scaling concepts

The dynamic scaling approach analyzes the spatial and temporal correlations of the surface height *h*(**r**, *t*) around its mean value 〈*h*〉^26^. This analysis is made through the study of the interface width or surface roughness, *w*(**r**, *t*), defined as *w*(**r**, *t*) = 〈(*h*(**r**, *t*) − 〈*h*〉)^2^〉^1/2^, where *t* is the deposition time and 〈..〉 denotes the average over all **r** in a system of lateral size *L*, with *r* ≤ *L*. Depending on the length scale in which *w* is computed, two important magnitudes are defined: (i) the global width or root-mean-square roughness, *σ*(*L*, *t*) = *w*(*L*, *t*) for *r* = *L*; and (ii) the local width, *w*(*r*, *t*), for *r* < *L*. In the standard description of kinetic roughening, both the global and the local widths follow the so-called Family–Vicsek (FV) dynamic scaling^26,27^,

1 $$w(r,t)={t}^{\alpha /z}\,f(r/{t}^{\mathrm{1/}z}),$$

where *f*(*u*) ~ *u* ^*α*^ for $u\ll 1$ and $f(u)\sim {\rm{constant}}$ for $u\gg 1$. Here, *α* is known as the roughness exponent and describes the spatial correlations of the surface, since *w* ∝ *r* ^*α*^ for very long times; it is thus related with the fractal dimension of the surface^26^. The critical exponent *z* is referred to as the dynamic exponent, as it describes the time increase of the typical lateral correlation length of the system, *ξ*, which is in many cases related to the grain size, as *ξ* ∝ *t* ^1/*z*^. Finally, the ratio *β* = *α*/*z* is known as the growth exponent, and it accounts for the roughening of the surface with time, since *σ* ~ *t* ^*β*^ for ${t}^{\mathrm{1/}z}\ll L$.

However, for some kinetically rough systems the roughness scales differently at global and local length scales^27,28,63,64^. Specifically, the global width still behaves as Eq. (1) but now the local width scales as

2 $$w(r,t)={t}^{\alpha /z}\,{f}_{A}(r/{t}^{\mathrm{1/}z}),$$

where ${f}_{A}(u)\sim {u}^{{\alpha }_{{\rm{loc}}}}$ for $u\ll 1$ and *f*_*A*_(*u*) ~ constant for $u\gg 1$. Accordingly, now for small distances *r* such that $r\ll \xi \ll L$, the roughness *w* scales as $w\sim {t}^{{\beta }^{\ast }}{r}^{{\alpha }_{{\rm{loc}}}}$, whereas for $r\gg \xi$, it scales as *w* ~ *t* ^*β*^, where *α*_loc_ and *β*_loc_ = *α*_loc_/*z* are the local roughness and growth exponents, respectively. In addition, β* = *β* − *β*_loc_ indicates the difference between the values of the global and the local growth exponents.

In order to assess the operating growth mode, it is necessary to analyze statistically the surface morphology at different growth times. Two main statistical functions are frequently^65,66^ employed. The first one is the Power Spectral Density (PSD), defined as *PSD*(*k*, *t*) = 〈*H*(**k**, *t*)(*H*(−**k**, *t*)〉, where *H*(**k**, *t*) is the space Fourier transform of *h*(**r**, *t*), and **k** is the spatial frequency in reciprocal space. The most general form for the radially-averaged PSD of isotropic, kinetically rough systems is discussed in ref.^64^, where up to four different growth scaling behaviors can be distinguished. For those relevant to our present work, the PSD behaves as

3 $$PSD(k,t)={k}^{-\mathrm{(2}\alpha +\mathrm{2)}}\,{f}_{P}(k{t}^{\mathrm{1/}z}),$$

where *f*_*P*_(*u*) ~ *u* ^2*α*+2^ for $u\ll 1$, and *f*_*P*_(*u*) ~ constant. for $u\gg 1$. Our experimental data will be seen to display up to two different scaling behaviors, namely, standard FV scaling with *α* = *α*_loc_ < 1, or super-rough scaling, where *α*_loc_ = 1 ≠ *α* > 1. The fact that a given system displays one type of scaling or the other reflects the physical mechanisms controlling the surface behavior and can be used to identify them. In general, non-FV scaling like super-roughness is regarded as non-standard or anomalous, being related with non-stationarity properties of the surface slopes^27,67^. These anomalous properties show up e.g. in the behavior of the height-difference correlation function, *G*_2_(*r*, *t*), which is a real-space correlation function known to scale as *w*(*r*, *t*)^26–28^, that is defined as

4 $${G}_{2}(r,t)={\langle {[h({\bf{r}}+{\bf{r}}{\rm{^{\prime} }},t)-h({\bf{r}}{\rm{^{\prime} }},t)]}^{2}\rangle }^{1/2}.$$

Specifically, in the presence of (super-rough) anomalous scaling^63,64,68^,

5 $${G}_{2}(r,t)\sim {r}^{{\alpha }_{{\rm{loc}}}}{t}^{{\beta }^{\ast }}\,{\rm{for}}\,r\ll {t}^{\mathrm{1/}z},$$

and

6 $${G}_{2}(r,t)\sim {t}^{\beta }\,{\rm{for}}\,r\gg {t}^{\mathrm{1/}z}\mathrm{.}$$

The two following properties will be useful below:

7 $${G}_{2}(r,t)/{r}^{\alpha }\sim {(r/{t}^{\mathrm{1/}z})}^{{\alpha }_{{\rm{loc}}}-\alpha }\,{\rm{for}}\,r\ll {t}^{\mathrm{1/}z},$$

and

8 $${G}_{2}(r,t)/{r}^{\alpha }\sim {(r/{t}^{\mathrm{1/}z})}^{-\alpha }\,{\rm{for}}\,r\gg {t}^{\mathrm{1/}z}\mathrm{.}$$

In order to identify different growth modes properly, it is interesting to note that *G*_2_(*r*, *t*) does not change with growth time at small distances for FV scaling (since *α* = *α*_loc_). This is not the case under super-roughening, when *α* > *α*_loc_ so that *G*_2_(*r*, *t*) curves shift upwards with time for small *r*. The different behaviors of the PSD and *G*_2_(*r*, *t*) with *k*, *r*, and *t*, are fingerprints to assess the actual growth mode.

## Results

**Figure 1:**
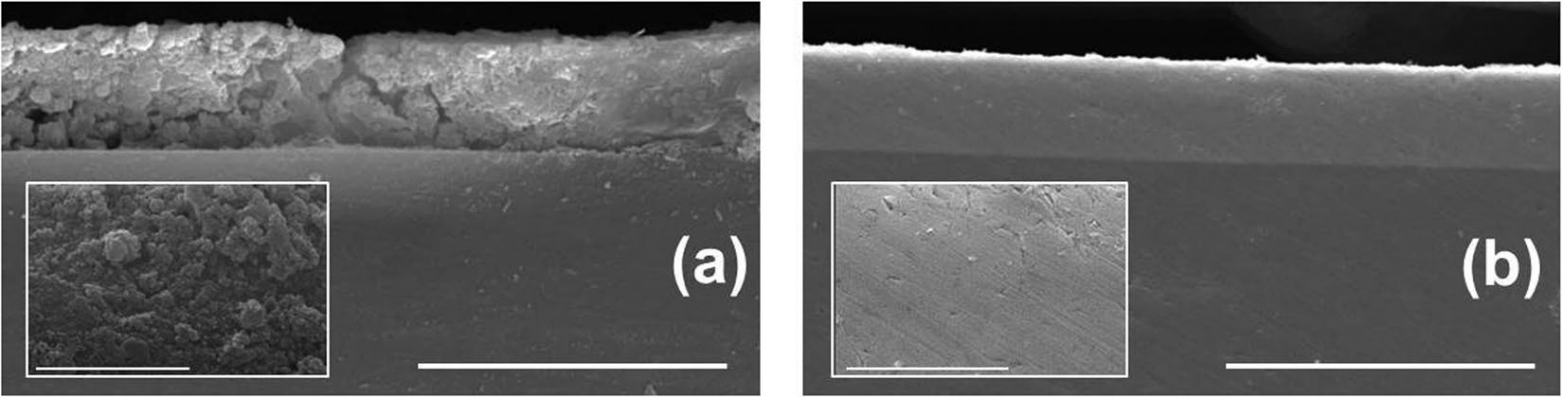
SEM images of the coating cross-sections for Ni (**a**) and NiW (**b**). The horizontal bars correspond to 20 *μ*m. The insets are higher magnification images in which the horizontal bars correspond to 3 *μ*m.

Figure 1a,b display characteristic SEM cross-section images of Ni and NiW films electrodeposited on steel substrates for 60 min. Clearly, the coating thickness is larger for Ni (close to 7.6 ± 1.2 *μ*m) than for NiW (6.7 ± 0.3 *μ*m). The insets display larger magnification images of the bulk of the films. The NiW film texture is quite compact and homogeneous, whereas the Ni film is porous and granular. These marked differences suggest that the corresponding growth processes may differ.

**Figure 2:**
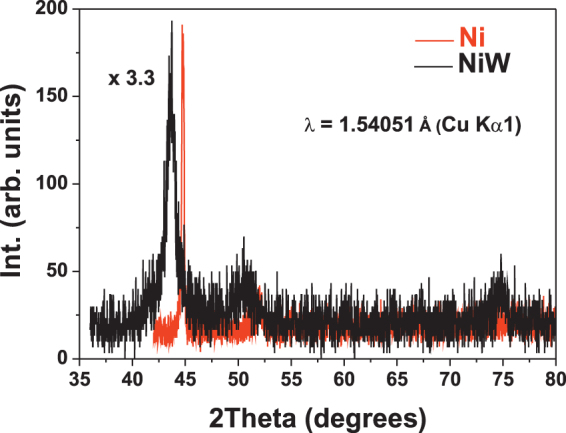
XRD diffraction pattern obtained for Ni (red) and NiW (black) films.

Figure 2 shows the XRD patterns obtained on the Ni and NiW films deposited for 80 minutes on steel substrates. Three peaks are clearly distinguished in the sampled angle range. Clearly, the NiW peaks shift towards lower angles with respect to Ni. This is due to the dissolution of W atoms in the fcc Ni lattice, which causes an expansion. The lattice constant can be obtained from the (111) reflection, yielding 0.352 nm and 0.359 nm for the Ni and NiW samples, respectively. From the lattice parameter of the NiW sample, the W content of the alloy can be determined using Vegard’s Law^69^. This calculation yields a W content close to 18% that matches quite well the EDS-SEM result (20%). Also, the crystallite size can be derived from the peak width using the Scherrer formula^70^. In this case, quite different sizes are obtained, namely, 33 nm for Ni and 9 nm for NiW, confirming differences in the growth mode of both systems.

**Figure 3:**
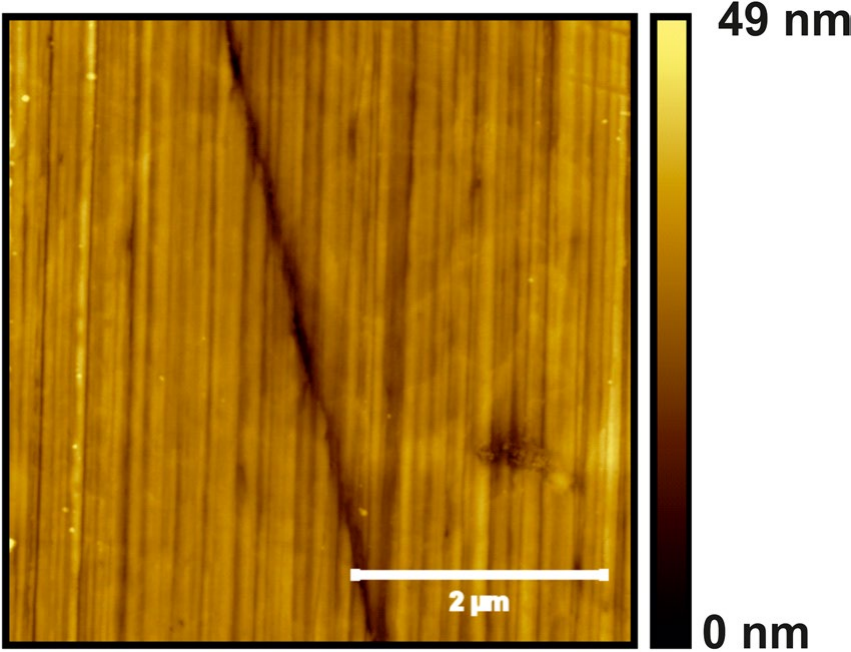
5 × 5 *μ*m^2^ AFM image of the polished steel substrate.

Figure 3 displays the morphology of the polished steel substrate. It is characterized by a series of parallel stripes separated by grooves. These structures show lateral widths of 70–160 nm and peak-to-valley height differences in the 5–9 nm range, the surface roughness being 2.9 nm. In addition, a wider (250 nm) and deeper (20 nm) groove is also observed, running in a different direction than the smaller ones.

**Figure 4:**
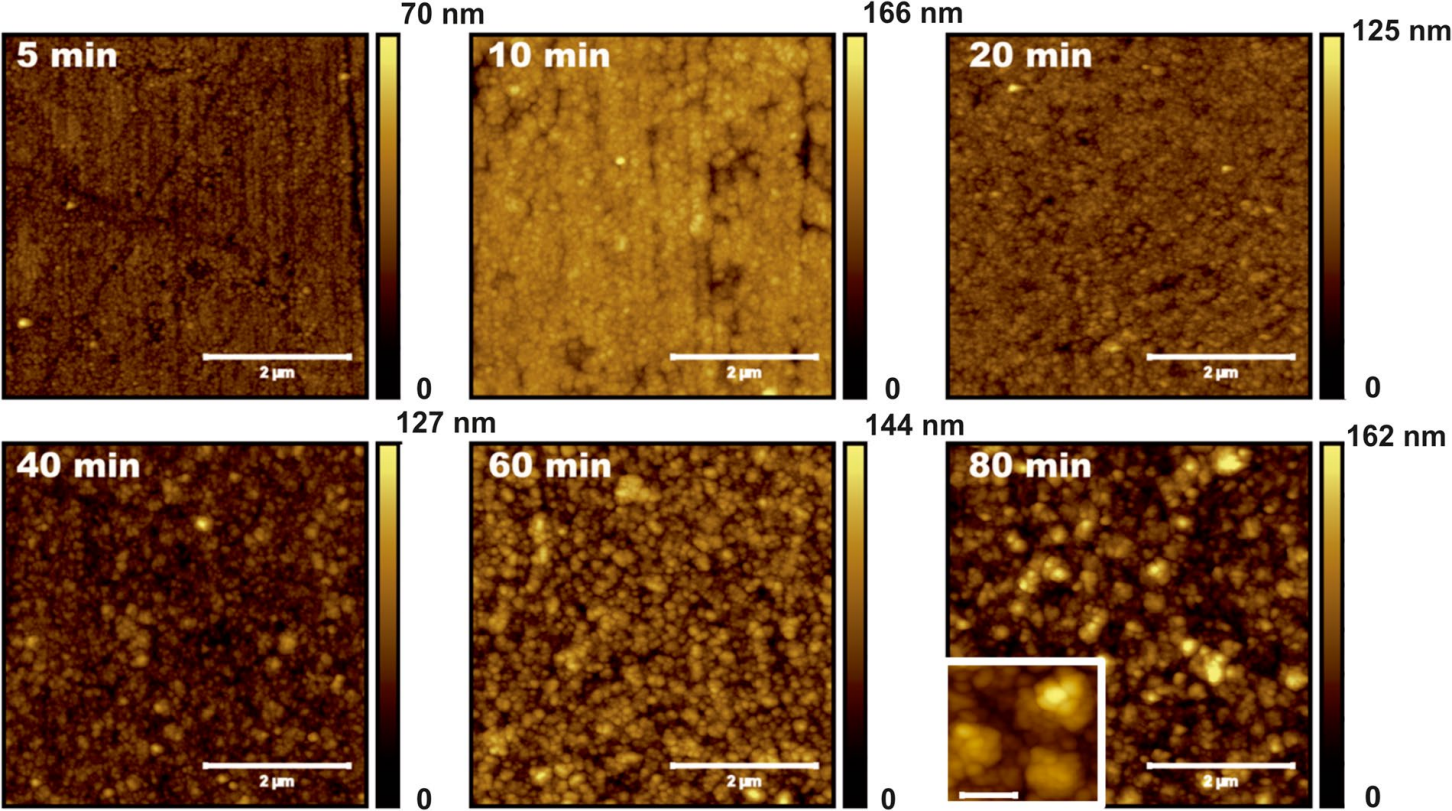
5 × 5 *μ*m^2^ AFM images of electrodeposited Ni films for times as indicated in each case. All scale bars represent 2 *μ*m. Inset of the last image: higher-resolution image (scale bar, 300 nm) for the same time, showing the cauliflower-like morphology.

The surface morphologies of Ni films electrodeposited on this substrate are shown in Fig. 4 for different times. Detailed inspection of the images reveals that the films have a granular structure whose size is in the 40–60 nm range, which is consistent with X-ray data taking into account tip convolution effects. Up to 20 minutes, a grooved morphology is still observed, likely reminiscent of the initial substrate morphology: for 5 and 10 minutes, parallel grooves are still visible, whereas for 20 minutes localized depressions remain. The persistence of the substrate morphology during the initial growth stages could be an indication that Ni does not “wet” easily the steel surface. After 40 minutes of ECD, the morphology becomes more homogeneous. Detailed analysis of the images shows that the granular structures aggregate leading to larger forms like those in the inset for 80 minutes. This type of structure is frequently referred to as cauliflower-like; it has been reported for films grown by many diffusion-limited techniques, like ECD and CVD^71,72^.

**Figure 5:**
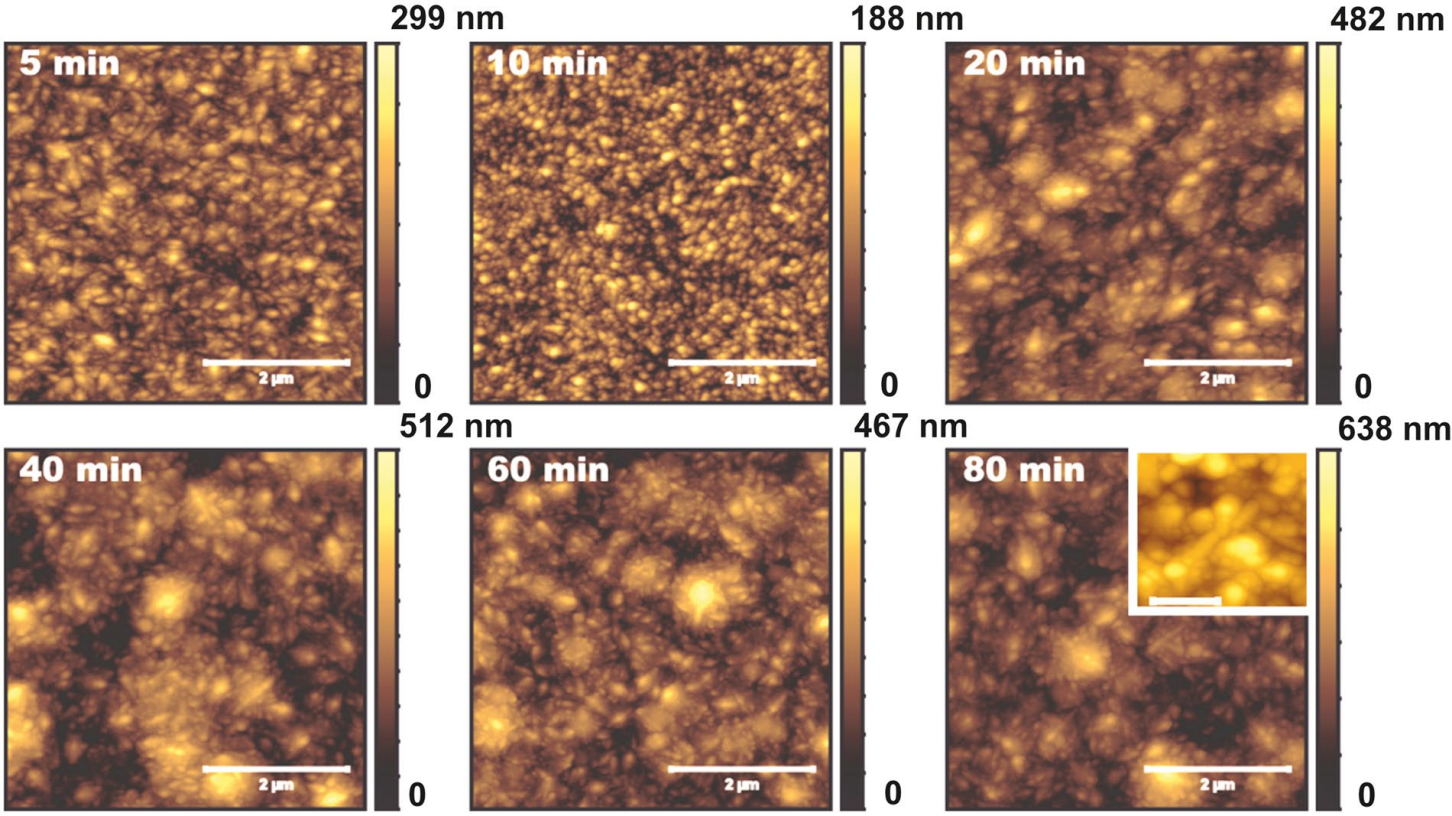
5 × 5 *μ*m^2^ AFM images of electrodeposited NiW films for times as indicated in each case. All scale bars represent 2 *μ*m. Inset of the last image: higher resolution image (scale bar, 300 nm) for the same time.

The film morphology is quite different for the NiW system, as can be observed in Fig. 5. The morphology is homogeneous already from the first stages of growth, with no traces of the substrate grooves. This indicates that the alloy covers more efficiently the steel surface. Elongated structures appear with random orientations, see the inset for 80 minutes. This morphology has been reported previously for NiW alloys using PC ECD^73^ and has been referred to as needle-like. Additional structures seem to agglomerate smaller ones, particularly after 20 minutes. Overall, there is no trace of the relatively round, cauliflower-like structures seen for the Ni films.

**Figure 6:**
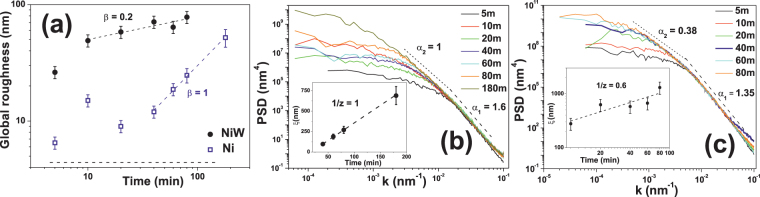
(**a**) Global surface roughness dependence with growth time for Ni (blue empty squares) and NiW (black solid bullets). Values of *β* correspond to the slopes of the oblique dashed lines. The horizontal dashed line indicates the roughness of the steel substrate. Radially-averaged PSD curves for different electrodeposition times for Ni (**b**) and NiW (**c**). The indicated values of *α* lead to power laws represented by the corresponding straight lines. The insets show the time dependence of the lateral correlation length, using linear/logarithmic axes for (**b**,**c**). Values of the coarsening exponent, 1/*z*, are indicated and provide the slopes of the dashed lines.

The qualitative morphological differences between both systems are quantitatively confirmed by the evolution of the roughness, see Fig. 6a. For Ni, *σ* is initially quite small, less than 7 nm after 5 min., and evolves non-monotonically for *t* < 40 min, likely due to the persistence of the grooved initial surface. As growth proceeds, Ni fills up depressions and *σ* is reduced. After 40 min., once the grooved morphology of the initial substrate does not seem to influence the film surface any longer, the roughness becomes almost linear with time: the behavior *σ* ~ *t* ^*β*^ with *β* = 1 is indicated in the figure for later reference, being related to the more homogenous film morphology and to the development of the cauliflower-like morphology^72^.

In contrast, for NiW the roughness is rather high already after 5 min., more than eight times that of the steel surface and almost four times that of the equivalent Ni case. At 10 minutes, *σ* has practically doubled its value at 5 min. In contrast, after 10 min. the roughness increases more slowly with time as *σ* ~ *t* ^*β*^, with *β* = 0.2 ± 0.05. Thus, the surface morphology for NiW becomes stabilized for long deposition times, as the roughening rate is appreciably slower than for Ni.

We can gain further insight into the surface space correlations through the analysis of the radially-averaged PSD functions for different deposition times, see Fig. 6b for Ni. The PSDs show two regions with non-trivial space correlations, one for large *k* values in which *PSD*(*k*) ~ *k* ^−5.2^ (i.e. *α*_1_ = 1.6) and another one for smaller *k*, with *PSD*(*k*) ~ *k* ^−4^ (i.e. *α*_2_ = 1). This second regime becomes particularly evident for the longest growth times. By defining the lateral correlation length, *ξ*, for each time as (the inverse of) the value of *k* separating *k*-independent from *k*-dependent behavior in the PSD, $\xi \sim {t}^{-\mathrm{1/}z}$ is expected to hold for kinetically rough surfaces^26^. The inset of Fig. 6b compares the *z* = 1 value expected for cauliflower-like morphologies^72^ to our experimental data. Note, as $\xi (t)\sim t$ in such a case, this behavior can be naturally assessed in a plot with linear (rather than logarithmic) axes, as chosen for this inset. The crossover between surface scales described by different roughness exponents in the PSD lies close to 0.0165 nm^−1^, which corresponds to a lateral distance of 60 nm. This value is close to the typical grain size measured by AFM. It is worth noting that the PSDs overlap for different times, indicating standard FV scaling^63,68^. However, *α*_1_ > 1 implies super-rough anomalous scaling at small time and length scales^63,65,68^.

Similar analysis yields qualitatively similar behavior for NiW, but with significant quantitative differences, see Fig. 6c. Two different scaling regimes are again observed. However, the large-*k* region now corresponds to (super-rough) power-law behavior with *α*_1_ = 1.35, a smaller roughness exponent than for Ni. Moreover, the crossover between large and small-*k* non-trivial behaviors shifts now to considerably larger distances, up to almost 190 nm. This value could be related to the size of the larger structures observed in the smaller images (see inset of Fig. 5). Finally, the most marked difference lies in the *α* value in the small-*k* region, which is considerably smaller than for Ni, as now *α*_2_ ≈ 0.38. With respect to time correlations, the inset of Fig. 6c indicates 1/*z* = 0.6 ± 0.2 in this large-scale range.

## Discussion

The results presented above evidence strong differences between the morphologies of the Ni and NiW electrodeposits. Both systems present two growth regimes, an initial (rather short for NiW) super-rough one with *α*_1_ > 1, followed by large-scale behavior with very strong fluctuations (*α*_2_ ≈ *β*_2_ ≈ 1/*z*_2_ ≈ 1) for Ni, or with much slower dynamics with *β*_2_ ≈ 0.2 and smaller roughness exponent, *α*_2_ ≈ 0.38, for NiW. For Ni (NiW) the change between short and large length-scale behaviors takes place around 50 nm (190 nm). This distance is relatively close to the measured crystallite size for Ni, but much larger than NiW crystallites. Note, super-rough anomalous scaling has been also assessed experimentally for *amorphous* films grown by ECD^74,75^.

**Figure 7:**
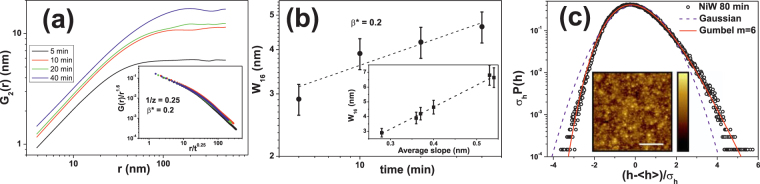
(**a**) *G*_2_(*r*) vs *r* for different electrodeposition times of Ni, as obtained on 1 × 1 *μ*m^2^ images. Inset: Collapse of the data in the main panel according to super-rough scaling. (**b**) Local surface roughness at *r* = 16 nm, *w*_16_, as obtained from (**a**), vs deposition time. Inset: *w*_16_ vs average slope. Note the direct proportionality. (**c**) Height probability distribution for NiW films at *t* = 80 min., normalized to zero mean and unit variance. Symbols are experimental data, the dashed magenta (solid red) line is a Gaussian (Gumbel, *m* = 6) distribution, *σ*_*h*_ is the root-mean square deviation of the height values, and 〈*h*〉 their space average. Inset: representative AFM top view image of a NiW surface after ECD for 80 min. The white bar represents 5 *μ*m and the full range of the height color bar corresponds to a 575 nm height difference.

We can further characterize the small-scale regime for Ni due to its relatively long duration. As expected, *α* > 1 induces a vertical shift of the *G*_2_(*r*, *t*) correlation functions for increasing growth times, see Fig. 7. This confirms the super-rough scaling hinted at by the PSD analysis (Fig. 6b). In principle, super-roughness implies *α*_loc_ = 1. However, we measure *α*_loc_ ≈ 0.8. As reported earlier^65^, this is due to a computational limitation of the *G*_2_(*r*, *t*) function, which typically underestimates *α*_loc_ in the presence of super-roughness (*α* > 1)^76^. Thus, we can assume *α*_loc_ = 1. The consistency of this scaling analysis can be further assessed by using Eq. (7). Thus, by plotting *G*_2_(*r*, *t*)/*r* ^*α*^ vs *r*/*t* ^1/*z*^, reasonable data collapse is indeed obtained at small arguments, see the inset of Fig. 7a, with scaling exponents β* = 0.20 and 1/*z* = 0.25. According to Eq. (7), the slope *m*_1_ of the master curve for small arguments should be *m*_1_ = *α*_loc_ − *α* ≈ −0.8, which indeed is the case using the measured *α*_loc_ ≈ 0.8, as elsewhere in the collapse. Note, we have restricted the collapse to *t* ≤ 40 min. Collapse at large arguments is not so good, due to the different growth regime that operates at large distances and long times.

One further check of the consistency of this analysis is based on the study of the evolution of the local roughness with time, when measured from Fig. 7a for a small *r* value (e.g. 16 nm, twice the nominal tip radius), see Fig. 7b. This plot is consistent with ${w}_{16}\propto {t}^{{\beta }^{\ast }}$ for β* = 0.20 ± 0.05, a value of the local growth exponent which agrees well, within the experimental errors, with the one obtained from the collapse of the *G*_2_(*r*, *t*) functions (Fig. 7a). It should be stressed that anomalous scaling is very frequently found in ECD experiments^54^ including the single available study of pulsed ECD^55^. This scaling behavior, being characterized by the upwards vertical shift of *G*_2_(*r*, *t*) with time, has been related with the time increase of the average surface slope^54,55,63,64,67,68,77^. Again, this is the case for our Ni films. When the *w*_16_ values are plotted vs the corresponding average slopes, obtained with the Gwyddion software^78^, proportionality clearly occurs, see inset of Fig. 7b. This is expected for a super-rough system, as *G*_2_(*r*, *t*) is known to be proportional to the surface slope for very small *r* ^27,67,76^. We have considered *r* = 16 nm, just twice the nominal tip radius, to avoid convolution effects.

The large-scale behavior for Ni is characterized by a morphological instability as, for long deposition *t* ≥ 20 min. after the film fully covers the substrate, the roughness increases linearly with time (i.e., *β*_2_ ≈ 1), associated with development of a cauliflower-like morphology with *α*_2_ ≈ 1. This type of surface and scaling exponents set have been also obtained for amorphous hydrogenated carbon films grown on silicon substrates by electron cyclotron resonance CVD^72^. They are theoretically predicted for growth systems with a high sticking probability for the depositing species and diffusion-limited transport, in which particles arrive to the growing surface following random trajectories^72^. Actually, the theoretical results in this reference allow to interpret the very small-*k* behavior of the PSD (see e.g. the 180 min. data with *k* < 0.003 nm^−1^) as a slow crossover between the *α*_2_ = 1 asymptotics and *k*-independent behavior for a finite system. Overall, this scenario is compatible with Ni ECD from a citrate bath. In fact, Ni can be deposited from different complexes, either with citrate or NH_3_, that can form in the electrolyte^5^. In addition, mass transport by bulk diffusion in the electrolyte is a characteristic mechanism in electrodeposition^54^. Moreover, our results also reveal that the relaxation time, *t*_OFF_, seems to be short enough to recover the concentration of electroactive Ni species near the electrode surface, thus driving the system to a diffusion-limited regime.

With respect to NiW, the initial anomalous regime (*α*_1_ > 1) lasts for a very short time, which prevents further study. We rather focus on the asymptotic regime, with exponents *α*_2_ = 0.38, *β*_2_ ≈ 0.2 ± 0.05, and 1/*z*_2_ = 0.6 ± 0.2. These values are actually consistent with Kardar-Parisi-Zhang (KPZ) scaling^39^, which implies conformal growth of surface features^26–28,79^. Furthermore, Fig. 7c provides the probability distribution function of height fluctuations, as experimentally obtained for *t* = 80 min. As expected for KPZ scaling, the height statistics are clearly non-Gaussian, note how the data deviate from the Gaussian distribution that has been included as a reference in the figure. In contrast with the 1D case, in which the Tracy-Widom distribution is analytically known^40,47,51^, this is not the case for 2D interfaces. Nevertheless, the statistics of height fluctuations is known for the latter to be quite approximately described by the Gumbel distribution *G*(*h*;*m*) with *m* = 6^46,53^. As also seen in Fig. 7c, this distribution does agree quite well with our experimental measurements. Note that no fit has been performed. In particular, the experimental height fluctuations feature values of skewness, *S* = 0.50 ± 0.05, and excess kurtosis, *K* = 0.38 ± 0.04, which are close to the numerical ranges which have been reported for two-dimensional surfaces in the KPZ class, in the so-called flat geometry case that applies here, i.e. *S*_2DKPZ_ = 0.412–0.435 and *K*_2DKPZ_ = 0.32–0.362^52,53^. Incidentally, the positive sign of the experimental skewness is as expected for a topography dominated by protuberances rather than by holes^80^.

Overall, the scaling behaviors that we find experimentally for Ni and NiW films can be largely understood within a single model of diffusion-limited film growth^59,60^. Accordingly, two main processes compete in techniques like CVD and ECD: mass transport (through the gas/electrolyte medium) and aggregation kinetics at the growing interface. Specifically, in the stochastic moving boundary problem put forward in^59,60^, the efficiency of the surface kinetics processes leading to eventual attachment of the depositing species are characterized by a kinetic rate constant *k*_*D*_ which can be related to the local sticking probability of individual molecules to the growing aggregate, see references in^59,60^. Then, depending on the relative importance of mass transport and surface kinetics, different film morphologies and growth dynamics are predicted. When the depositing species incorporate relatively fast to the growing aggregate, the process becomes limited by mass transport. This behavior (which we will denote as a high sticking-probability condition) is described through a rate of metal incorporation, *k*_*D*_, which is much larger than the average growth velocity. In such a case, protruding surface regions receive more depositing particles than surface depressions (geometrical shadowing) leading to unstable growth, since local height variations become amplified. This scenario has been shown to lead to cauliflower-like morphologies and scaling exponents *α* = *β* = 1/*z* = 1^72^, compatible with our present observations for Ni films at long times. Mass-transport-dominated systems like this are in fact quite standard in ECD^54^.

In the opposite situation, in which the reaction kinetics at the surface is inefficient (corresponding to a small *k*_*D*_ value; low sticking probability condition), the growth dynamics becomes limited by attachment. In this case, the model predicts^59,60^ an asymptotic KPZ growth mode, namely, surface growth is essentially conformal^26–28,79^, but with a roughness that fluctuates substantially in space and time. Note, KPZ scaling has been very scarcely observed in electrodeposited systems^56^ as it is not easy to tune the experimental systems to conditions with such low sticking probabilities. Nevertheless, in our NiW system asymptotic KPZ scaling is indeed observed. Therefore, we have to consider whether a scenario with a low sticking probability of the depositing species is consistent with NiW electrochemical co-deposition: Electrochemical induced co-deposition is a rather complex system in which both, kinetic and transport mechanisms, are coupled. Thus, many arguments have been proposed to understand the mechanism of induced co-deposition of alloys, e.g. for W with iron-group metals in citrate or citrate&ammonium electrolytic solutions, see a recent review in^81^. Models can be roughly classified as either complexing-species models^5^ or adsorption models^81–84^. The latter consider growth processes in which hydrogen or intermediates hinder growth by adsorbing at the film interface. A depositing species that arrives at such blocked sites will not remain there but will, rather, visit different interface locations until finally reaching an available site. Statistically, this situation implies a low sticking probability. KPZ asymptotic scaling can be thus explained for the NiW system. Moreover, the low-sticking condition also agrees with the enhanced compactness of the NiW film (Fig. 1), as compared with the more porous structure of the Ni film. When the sticking is low the incoming species can aggregate both at surface protrusions and depressions while, under high sticking conditions, they attach mainly at the former, leading to a more porous structure. Furthermore, lower sticking for NiW is also consistent with a smaller film thickness, as observed.

It should be noted that a rather similar behavior was reported by some of the present authors for CVD deposition of silica films on Si substrates^85^. In that study, asymptotic KPZ scaling was also found for low-sticking conditions and unstable growth dynamics took place for conditions governed by mass transport. In that case, the sticking probability was controlled by the growth temperature. Indeed, to some extent film growth by CVD and by ECD are conceptually similar^59,60^. In both cases, the depositing species form in the bulk of a dilute phase and diffuse across it to reach the film interface, where eventual attachment is mediated by chemical reactions with a finite efficiency. These similarities are made explicit through the continuum evolution equation proposed in^85^, which is essentially valid for our electrodeposition systems^59,60^, namely,

9 $${\partial }_{t}h({\bf{r}},t)=\nu {\nabla }^{2}h-{\mathscr{K}}{\nabla }^{4}h+\varepsilon \theta /\langle \theta \rangle +\lambda {(\nabla h)}^{2}+\eta \mathrm{.}$$

**Figure 8:**
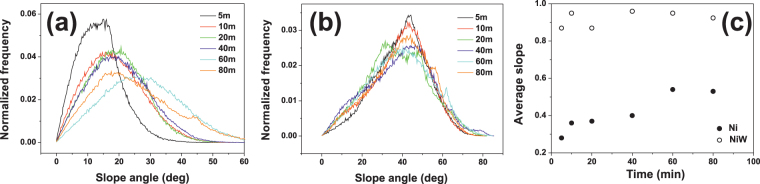
Slope angle distributions for different electrodeposition times for Ni (**a**) and NiW (**b**). Panel (c) shows the time dependence of the average slope, as obtained from the distributions in (**a**,**b**).

Here, ∂_*t*_ *h* is the local height velocity, *η* is a stochastic noise term accounting for the random arrival and sticking of the depositing species, and values for the parameters *ν* > 0, ${\mathscr{K}} > 0$, *ε*, and *λ* depend on experimental conditions. The first term on the right-side of Eq. (9) describes surface smoothening through evaporation/condensation processes; the second term is related to relaxation through surface diffusion mechanisms; the third term represents geometric shadowing effects, with *θ* being the local exposure angle and 〈*θ*〉 its space average, while the fourth term represents (conformal^79^) growth along the local normal direction, and is a landmark of KPZ scaling^26–28,39^. When growth is limited by mass transport, geometric shadowing dominates, leading to unstable morphologies which are predicted by Eq. (9) using relatively large values of *ε* ^85^. In contrast, growth which is limited by surface kinetics corresponds to small *ε* ^85^, in which case the KPZ nonlinearity dominates at large length scales and long times, as observed for NiW films. Note that the KPZ term becomes significant when the local slopes are sufficiently large. Therefore, an important check of the consistency of the KPZ scaling in the NiW system is to analyze its surface slope distribution and compare with Ni, for which the KPZ term does not seem to operate significantly. This analysis is shown in Fig. 8, which indeed shows strong differences between the two films. For Ni, the slope distribution is asymmetric, with a long tail in the high-slope range and a most probable value around 15°–25° which increases with electrodeposition time (Fig. 8a). In contrast, for NiW the slope distribution does not change substantially with time. It is centered around 45° and is more symmetric, with a tail for low angles (Fig. 8b). From these distributions, the average slope values can be plotted for both systems as a function of time (Fig. 8c).

The average slope increases with time for Ni, in agreement with the super-rough anomalous behavior (Fig. 7b, inset). For NiW, the average slope is considerably larger already from the very first stages of growth: after 10 minutes, when the KPZ scaling regime starts, the average slope is close to 0.95 (slope angle ≈ 43°), three times its value for Ni (0.36, implying slope angle ≈ 20°). However, such a slope value remains virtually unchanged with time. This is consistent with KPZ-dominated dynamics^86^. Again, recall that KPZ scaling is characteristic of conformal growth^26–28^, the origin of its characteristic nonlinearity being growth with a constant rate along the local normal direction^39,79^.

Let us further note that KPZ scaling appears linked to alloy induced co-deposition, but not necessarily to the use of a complexing agent, since the latter is also employed for Ni growth which undergoes, rather, unstable growth dynamics. Moreover, the occurrence of KPZ scaling in the NiW system does depend on the operating conditions, particularly on the current density: When considerably higher currents were employed for NiW, a cauliflower-like morphology was obtained^15^, whose dynamics is again controlled by mass transport^87^. This is again in agreement with the theoretical predictions^59,60^ for a system where the attachment kinetics is faster than mass transport.

## Conclusions

We have electrodeposited Ni and NiW films on polished steel substrates from citrate & ammonia plating baths under pulsed galvanostatic conditions. SEM characterization shows that NiW films are thinner and more compact than Ni films. The surface growth dynamics was studied by *ex*-*situ* AFM and the data were analyzed under the framework of kinetic roughening, well-suited for highly disordered surfaces like these. Both systems present an initial super-rough growth behavior which changes for long times into a different, asymptotic growth regime. The main differences between both systems are found in this long-time behavior. While for Ni we obtain rough interfaces compatible with *α*_2_ = *β*_2_ = 1/*z*_2_ = 1, corresponding^72^ to the cauliflower-like morphology assessed by AFM, for NiW a nodular morphology is observed, characterized by scaling behaviour consistent with a KPZ growth mode. Hence, the long-time evolution of the NiW film is morphologically stable, in contrast with the Ni system, and to our knowledge provides the first confirmation of KPZ asymptotics (including exponent values and height statistics) for 2D surfaces grown by ECD. The differences in the morphological behavior are explained by the different physical mechanisms which control growth (i.e., by the slowest process) in each system. In the Ni case, growth is limited by mass transport through the electrolyte, while for NiW the slowest process is the final deposition of the incoming species to the growing interface, due to a more inefficient surface kinetics. These results agree with a theoretical model of thin film growth by diffusion-limited techniques, like CVD and ECD, in which the key parameter is the relative importance of mass transport with respect to the kinetics of the attachment reaction. Our findings show that the slow surface metal incorporation that can occur via suitable induced co-deposition conditions is able to enhance conformal growth, leading to electrodeposition of more compact, albeit somewhat rougher, thin films.

## Methods

We have performed the electrodeposition of micro-nanocrystalline Ni–W deposits in the PC mode, since this procedure improves current distribution and mass transport processes controlling composition and microstructure of the alloy^14,15^. The composition of each component of the alloy can be controlled through the current density and the composition of the electrolytic solution. Plating baths containing citrate have been used in these experiments due to their effectiveness to obtain smooth, compact, and adherent deposits with controllable thickness. Citrate, like other organic acids, forms soluble complexes with Ni^2+^ and (WO_4_)^−2^ ions and avoid hydroxide formation^81^. In addition, we have employed ammonium salts in the electrolytic bath to regulate the pH and to improve Faradaic efficiency.

Ni–W coatings were obtained by pulsed galvanostatic ECD on 1 cm^2^ area carbon steel (SAE 1020) plates, which were previously polished with grit paper in decreasing size from 80 to 2500, followed by 0.3 *μ*m alumina powder and washed with Milli-Q water. The pulse program was performed by a TEQ electrochemical equipment and consisted in a 5 ms cathodic galvanostatic pulse of 40 mA/cm^2^ followed by a null current step applied for the same time. These conditions were kept fixed and only the electrodeposition time was varied in the 5–80 min. range. The plating bath contained 0.06 M NiSO_4_·6H_2_O, 0.14 M Na_2_WO_4_·2H_2_O, 0.5 M Na_3_C_6_H_5_O_7_·2H_2_O, 0.5 M NH_4_Cl, and 0.15 M NaBr (pH 9.5). For Ni electrodeposits (without W) the composition of the solution was the same except for the absence of Na_2_WO_4_·2H_2_O. A fresh plating bath was used in each experiment and pure chemical reagents and Milli-Q water were employed. The solutions were gently stirred during ECD and were deaerated by bubbling purified N_2_ gas. The temperature of plating bath was 65 °C.

Thickness measurements from cross section analysis of the Ni-W coatings and chemical composition of all samples were analyzed using an scanning electron microscope (SEM) FEI, Quanta 200 equipped with an energy dispersive spectroscopy (EDS) detector. The samples were characterized by Atomic Force Microcopy (AFM) using a Nanoscope IIIa system, operating in the dynamic mode in air conditions. Silicon tips (nominal force constant of 40 N/m from Bruker) with a nominal radius of curvature of 8 nm were employed. Images of *L* = 1, 2, 5, and 16 microns were measured at different spots of the surface and were composed by 512 × 512 pixels. For the thickest NiW films, images with scan size up to 50 *μ*m were also obtained. The statistical analysis of the AFM data has been made using the Gwyddion free-software^78^ and custom-made numerical routines. Additionally, XRD diffraction patterns were obtained using a D8 Bruker 4-circles diffractometer using Cu K_*α*1_ radiation.
